# Cerebrospinal fluid circulating tumour DNA genotyping and survival analysis in lung adenocarcinoma with leptomeningeal metastases

**DOI:** 10.1007/s11060-023-04471-8

**Published:** 2023-10-28

**Authors:** Kaixuan Bai, Xin Chen, Xuejiao Qi, Yu Zhang, Yueli Zou, Jian Li, Lili Yu, Yuanyuan Li, Jiajia Jiang, Yi Yang, Yajing Liu, Shuanghao Feng, Hui Bu

**Affiliations:** 1 Department of Neurology, The Second Hospital of Hebei Medical University, 215 Heping West Road, Xinhua District, Shijiazhuang, Hebei Province China; 2 The Key Laboratory of Neurology (Hebei Medical University), Ministry of Education, Shijiazhuang, China; 3 Neurological Laboratory of Hebei Province, Shijiazhuang, China; 4 Department of Neurology, Xingtai People’s Hospital, Xingtai, China; 5 Department of General Practice, Hengshui People’s Hospital, Hengshui, China

**Keywords:** Cerebrospinal fluid, ctDNA, Leptomeningeal metastases, Lung adenocarcinoma, Prognosis

## Abstract

**Purpose:**

The prognosis of patients with leptomeningeal metastasis (LM) remains poor. Circulating tumour DNA (ctDNA) has been proven to be abundantly present in cerebrospinal fluid (CSF); hence, its clinical implication as a biomarker needs to be further verified.

**Methods:**

We conducted a retrospective study of 35 lung adenocarcinoma (LUAD) patients with LM, and matched CSF and plasma samples were collected from all patients. All paired samples underwent next-generation sequencing (NGS) of 139 lung cancer-associated genes. The clinical characteristics and genetic profiling of LM were analysed in association with survival prognosis.

**Results:**

LM showed genetic heterogeneity, in which CSF had a higher detection rate of ctDNA (*P* = 0.003), a higher median mutation count (*P* < 0.0001), a higher frequency of driver mutations (*P* < 0.01), and more copy number variation (CNV) alterations (*P* < 0.001) than plasma. The mutation frequencies of the *EGFR, TP53, CDKN2A, MYC and CDKN2B* genes were easier to detect in CSF than in LUAD tissue (*P* < 0.05), possibly reflecting the underlying mechanism of LM metastasis. CSF ctDNA is helpful for analysing the mechanism of EGFR-TKI resistance. In cohort 1, which comprised patients who received 1/2 EGFR-TKIs before the diagnosis of LM, *TP53 and CDKN2A* were the most common *EGFR*-independent resistant mutations. In cohort 2, comprising those who progressed after osimertinib and developed LM, 7 patients (43.75%) had *EGFR* CNV detected in CSF but not plasma. Furthermore, patient characteristics and various genes were included for interactive survival analysis. Patients with *EGFR*-mutated LUAD (*P* = 0.042) had a higher median OS, and CSF ctDNA mutation with *TERT* (*P* = 0.013) indicated a lower median OS. Last, we reported an LM case in which CSF ctDNA dynamic changes were well correlated with clinical treatment.

**Conclusions:**

CSF ctDNA could provide a more comprehensive genetic landscape of LM, indicating the potential metastasis-related and EGFR-TKI resistance mechanisms of LM patients. In addition, genotyping of CSF combined with clinical outcomes can predict the prognosis of LUAD patients with LM.

**Supplementary Information:**

The online version contains supplementary material available at 10.1007/s11060-023-04471-8.

## Introduction

Leptomeningeal metastasis (LM) is a severe complication associated with metastatic solid tumours that is caused by neoplastic cells transferring to the pia mater, arachnoid, and subarachnoid space [1]. The incidence of LM is highest in melanoma, followed by advanced lung cancer, accounting for 3–5% [2]. Based on 2019 global cancer data, non-melanoma skin cancer, tracheal, bronchus, and lung (TBL) cancer, and colon and rectal cancer have the highest morbidity rates. Meanwhile, TBL cancer showed the highest mortality rate, with an age-standardized death rate of 25.18 [3].The incidence of LM in non-small cell lung cancer (NSCLC) patients is 3.4%, and that in *EGFR*-mutant patients is 9.4% [4]. The prognosis of patients with LM remains poor, with a median survival of less than 1 year despite treatments such as chemotherapy and whole-brain radiation therapy [5]. Since the first experiments targeting epidermal growth factor receptor (*EGFR*), targeted therapy has begun a new chapter based on an actionable molecular alteration in lung cancers [6]. Unfortunately, the vast majority of advanced NSCLC patients become resistant to current targeted treatments and eventually progress [7].

It is essential to characterize genomic diversity between primary and metastatic cancers. Nevertheless, tissue biopsies are invasive procedures, and some locations are not easy to access, such as the bones and brain [8]. However, tissue biopsy of LM is impractical due to diffuse distribution. On various occasions, circulating tumour DNA (ctDNA) is easier to collect serially than tissue biopsy. For patients who are harbouring difficult-to-biopsy neoplasms, such as pia mater or pia mater, ctDNA can provide critical molecular profiling and precision medicine treatment response in real time [9–11]. Alix-Panabières et al. elucidated that the detection and monitoring of ctDNA can provide effective information concerned with treatment decisions in cases of targeted therapy or immune checkpoint inhibition (ICI). ctDNA can be used to monitor the effect of targeted therapy and is a more suitable biomarker for liquid biopsy monitoring [8].

Due to the existence of the blood‒brain barrier (BBB), genotyping of the primary tumour and plasma does not represent the genetic alterations of cerebrospinal fluid (CSF) in LM. Currently, CSF is considered a crucial liquid biopsy medium for LM that provides an accessible and less invasive method to acquire information on genomic alterations [12]. CSF ctDNA exhibits unique genetic profiles and distinct resistance mechanisms during treatment in NSCLC patients with brain metastases (BMs), so it is crucial to test for acquired resistance at CNS progression [13, 14]. ctDNA is more abundantly present in the CSF than in plasma and has been shown to be a biomarker of treatment effect and tumour evolution in patients with LM [15–17]. To fully explore the effectiveness of CSF ctDNA representing genetic information in LM versus that of plasma ctDNA in our study, we compared the matched CSF and plasma from the same LM patients. However, few studies have analysed the correlation between ctDNA of CSF and/or plasma and survival outcome in patients with LM. In this study, we investigated the outcome of lung adenocarcinoma (LUAD) patients with LM in the real world and analysed the factors associated with their survival.

## Materials and methods

### Study design and patients

In this retrospective cohort study, we collected data from LUAD patients with LM who were enrolled in the Department of Neurology, The Second Hospital of Hebei Medical University (Hebei, China), from December 2015 to December 2022. The inclusion criteria were as follows: (1) > 18 and ≤ 75 years old; (2) diagnosis of LM according to the European Association of Neuro-Oncology-European Society for Medical Oncology (EANO-ESMO) clinical practice guidelines [18]; and (3) all LM patients underwent lumbar puncture. The exclusion criteria were as follows: (1) excessive missing clinical data; and (2) incomplete follow-up information. ctDNA was extracted from CSF and plasma samples and detected by a 139-gene next-generation sequencing (NGS) panel at the time of diagnosis of LM(Table S1). Finally, a total of 35 patients with LM were included for further analysis. Demographic, clinicopathological and therapies data were obtained for each patient. The study was approved by the Research Ethics Committee of The Second Hospital of Hebei Medical University. Included patients from our institute provided signed informed consent. This study was conducted in accordance with the principles of the Declaration of Helsinki.

To study clinical outcomes, overall survival (OS) was measured. OS was defined as the time from LM to death or last follow-up.

### Preparation of CSF ctDNA, and plasma ctDNA

Freshly frozen CSF, and whole blood samples were collected for genomic profiling. Total DNA from freshly frozen CSF and plasma was extracted by QIAamp Circulating Nucleic Acid Kit (Qiagen, Germantown, MD, USA).

### NGS library preparation and sequencing data analysis

The Illumina libraries were carried out with KAPA Hyper Prep Kit (Kapa Biosystems, Woburn, MA, USA) according to the manufacturer’s protocol. Targeted panel sequencing was performed by SeqCap EZ System (Roche Nimblegen, Madison, WI, USA), and obtained by the Geneseeq Prime™ panel (Nanjing Geneseeq Technology Inc., Nanjing, JiangSu, China) which was covering 139 cancer-associated genes. On HiSeq X10 sequencing system (Illumina, San Diego, CA, USA), enriched libraries were sequenced with 150 bp pair-end reads.

### Statistical analysis

Survival analysis was performed using Kaplan‒Meier method with log-rank P values and 95% confidence intervals (CIs) reported. All *P* values were 2-sided and were considered statistically significant at *P* < 0.05. Multivariate analysis was performed using Cox proportional hazard regression with inclusion of variables significant on univariate regression.

Associations between two variables were analyzed using Fisher’s exact, Chi-square tests or Wilcoxon test. Graph Pad Prism version 9.0, SPSS version 25.0 and R version 4.1.2 software used for statistical analyses and making graphs.

## Results

### Patient characteristics

We retrospectively profiled 35 LUAD patients with LM for analysis. All patients had matched CSF and plasma samples. The demographic and clinical characteristics of the included patients are shown in Tables 1 and S2. The majority of the patients denied a smoking history (77.14%), 18 patients (51.43%) were males, and the median age was 54, ranging from 30 to 75 years. *EGFR* mutations were found in 26 patients (74.29%) at the initial diagnosis of LUAD. Among them, 13 patients (37.14%) were diagnosed with LM and BMs simultaneously, and 23 patients (65.71%) had extracranial metastases. Most of the patients suffered from neurologic symptoms prior to LM diagnosis. The most common clinical manifestations were cerebral symptoms (97.14%), such as headache and dizziness. Posterior fossa symptoms, including various cranial neuropathies (51.43%) and spinal cord/root symptoms, such as motor and sensory dysfunction (13%), were also in evidence. Thirty-three patients (94.29%) received targeted therapy and other therapy combinations (angiogenesis inhibitors/ICIs/chemotherapy/radiotherapy). All patients underwent intrathecal methotrexate via lumbar puncture or Ommaya reservoir. Thirty-two patients (91.43%) displayed malignant cells in CSF. The diagnosis of LM was established by CSF cytology alone in 10 patients (28.57%) and by both MRI and CSF cytology in 21 patients (60%). LM developed in 33 patients (94.29%) during the course of lung cancer, and LUAD was detected in 2 patients (5.71%) at the time of LM diagnosis. CSF intracranial pressure was decreased (≤ 200 mmH_2_O) in 57.14% of patients with LM. CSF abnormal biochemical measures also occurred in some LM patients.

**Table 1:** Patient demographic and clinical Characteristics

Characteristic	N (%)
Age at diagnosis	
Median (range)	57 (38–75)
< 60	19 (54.29)
≥ 60	16 (45.71)
Gender	
Male	18 (51.43)
Female	17 (48.57)
Smoking status	
Current/former smoker	8 (22.86)
Non‐smoker	27 (77.14)
EGFR-mutated LUAD	
Yes	26 (74.29)
No	9 (25.71)
KPS	
< 70	15 (42.86)
≥ 70	20 (57.14)
ECOG PS	
< 2	5 (14.29)
≥ 2	30 (85.71)
Median time interval between diagnosis of primary tumors and LM(months)	
Median (range)	21.5 (0–97)
≤ 22	23 (65.71)
> 22	12 (34.29)
Concurrent brain metastases	
Yes	13 (37.14)
No	22 (62.86)
Extracranial organ metastases	
Yes	23 (65.71)
No	12 (34.29)
Cerebral symptoms	
Yes	34 (97.14)
No	1 (2.86)
Posterior fossa symptoms	
Yes	18 (51.43)
No	17 (48.57)
Spinal cord/root symptoms	
Yes	15 (42.86)
No	20 (57.14)
Treatments before the diagnosis of LM	
Local treatment^a^	1 (2.86)
Systemic therapy^b^	17 (48.57)
Local treatment + Systemic therapy	14 (40)
Native	2 (5.71)
NA	1 (2.86)
Treatments at the time of LM diagnosis	
Targeted therapy	1 (2.86)
Targeted therapy + Other therapy^c^	33 (94.29)
Angiogenesis inhibitors + ICIs	1 (2.86)
Intrathecal methotrexate	35 (100)

^a^Radiotherapy/Surgery

^b^Targeted therapy /Angiogenesis inhibitors/ICIs/Chemotherapy

^c^Angiogenesis inhibitors/ICIs/Chemotherapy/Radiotherapy

KPS: karnofsky performance scale, ECOG PS: eastern cooperative oncology group performance score, ICIs: immune checkpoint inhibitors

### Genetic divergence of CSF and plasma samples

**Fig. 1:**
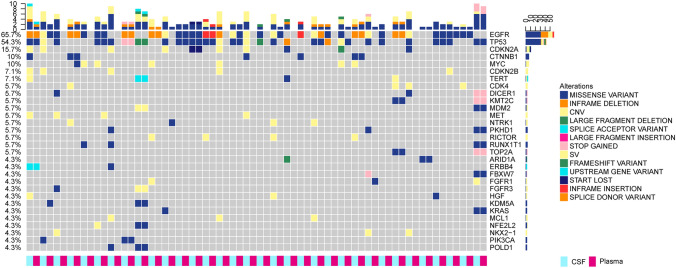
Genetic landscape of 35 LM patients. All patients matched CSF and plasma ctDNA. One patient was not shown, because no somatic mutation was detected in the CSF and plasma. These top bar display the number of genetic alterations of each patient, these right side-bar indicate the mutated genes, and these left side-bar present the percentage of genetic alterations. LM: leptomeningeal metastasis, ctDNA: circulating tumor DNA, CSF: cerebrospinal fluid

**Fig. 2:**
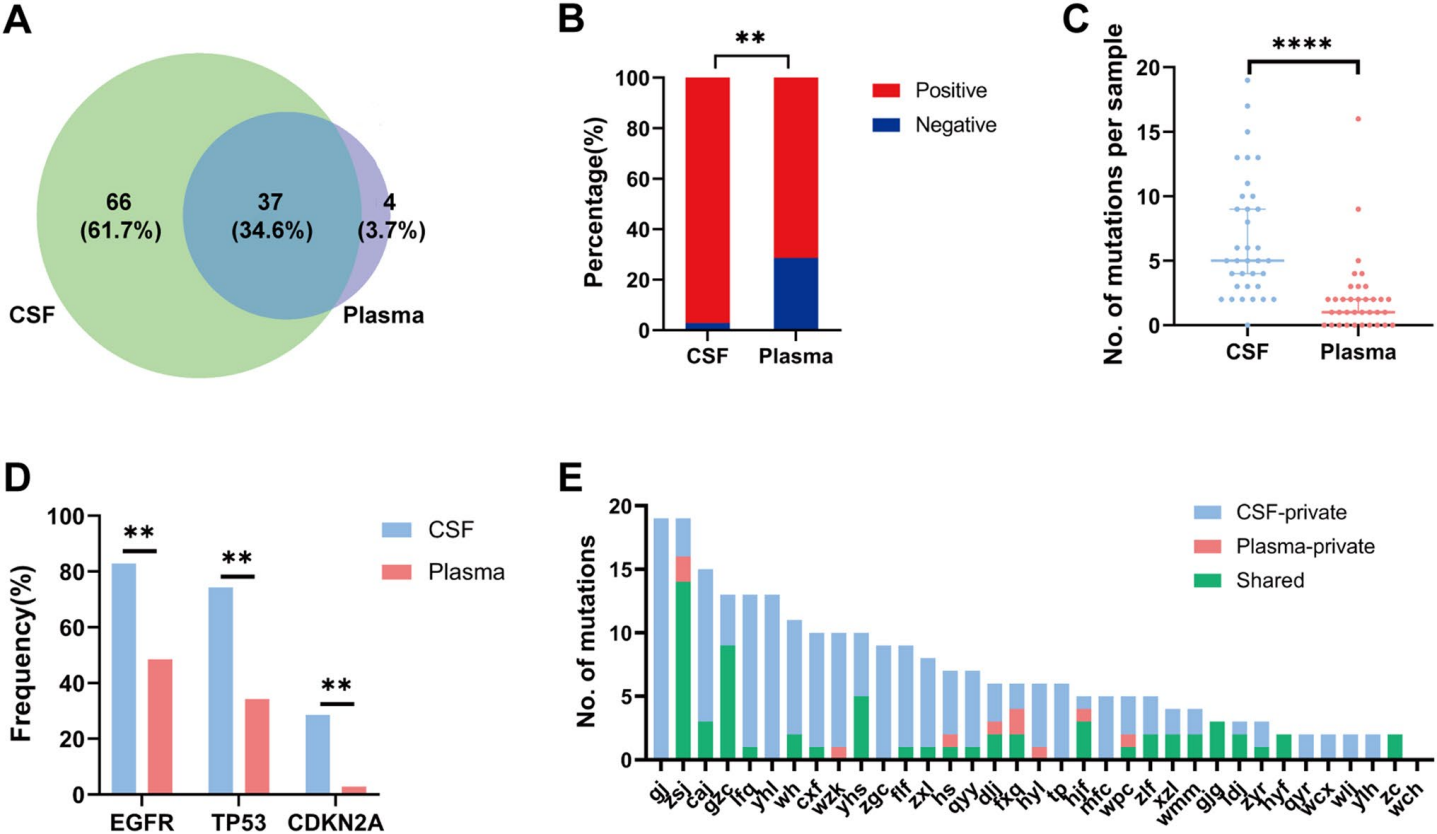
CSF and plasma ctDNA molecular characterization. **A**: venn diagram of mutated genes between CSF and plasma ctDNA analysis. **B**: the detection rate of ctDNA is statistically different between CSF and plasma. **C**: comparison of mutations count per sample between CSF and plasma. **D**: mutation frequency analysis of genes between CSF and plasma. **E**: frequency of CSF-private or plasma-private mutations versus shared mutations in paired CSF-plasma samples. CSF: cerebrospinal fluid; ctDNA: circulating tumor DNA. *P < 0.05, **P < 0.01, ***P < 0.001, and ****P < 0.0001

To delineate the genetic landscape, the somatic mutations from the matched CSF and plasma ctDNA of 35 patients were analysed by applying an NGS panel (Fig. 1**)**. No somatic mutation was detected in the CSF and plasma of one patient. A total of 262 somatic variants of 103 mutated genes were detected in 35 CSF samples. Altogether, 85 somatic variants of 41 mutated genes were identified in 35 plasma samples, presenting a lower count than in CSF. For the detection of genomic alterations, only 37 (34.6%) were present in both plasma and CSF. However, 66 (61.7%) could be detected in CSF but could not be found in plasma, while 4 (3.7%) could be found in plasma but could not be detected in CSF (Fig. 2A). ctDNA was positive in 34 patients (97.14%) in CSF and in 25 patients (71.43%) in plasma, and the detection rate of ctDNA was significantly different in CSF and plasma (*P* = 0.003, Fig. 2B). The median mutation count of CSF was 5, which was significantly greater than that of plasma ctDNA (5 vs. 1, *P* < 0.0001, Fig. 2C). *EGFR* (29/35, 82.86%), *TP53* (26/35, 74.29%) and *CDKN2A* (10/35, 28.57%) mutations frequently occurred in CSF, and coexisting *TP53* and *EGFR* were found in 22 (22/35, 62.9%) CSF samples. In addition, the most frequently altered genes in plasma were *EGFR* (17/35, 48.57%) and TP53 (12/35, 34.29%). Based on statistical analysis, the mutation frequencies of *EGFR, TP53* and *CDKN2A* were significantly higher in CSF than in plasma (*P* < 0.01, Fig. 2D). In addition, *EGFR* was the most frequent copy number variation (CNV) alterations in CSF, followed by *CDKN2A* (6/35, 17.1%) and *MYC* (6/35, 17.1%) mutations. However, only one CNV alteration was detected in the plasma sample. The percentage of CSF variants with CNV alterations was higher than that of plasma (83/262, 31.68% vs. 1/85, 1.18%, *P* < 0.001). Therefore, these results suggested that CSF had higher CNV alterations, and the importance of CNV in these alterations needs to be explored.

To further evaluate the genetic relevance and divergence between CSF and plasma, we analysed the shared and independently evolved mutations in each individual. In the 35 paired CSF-plasma pairs, a median 14.29% shared mutation rate was seen, which was relatively low. There were a few shared mutations found in 65.71% (23/35) of paired samples (Fig. 2E). Overall, LM showed genetic heterogeneity with slight differences in mutational prevalence between CSF and plasma. Plasma ctDNA was not reflective of LM status. All these data demonstrated a result of the low sensitivity of mutation detection via plasma biopsy. CSF might be a better ctDNA method to detect mutations of the tumour than liquid biopsy.

### The potential metastatic mechanism of LM

**Fig. 3:**
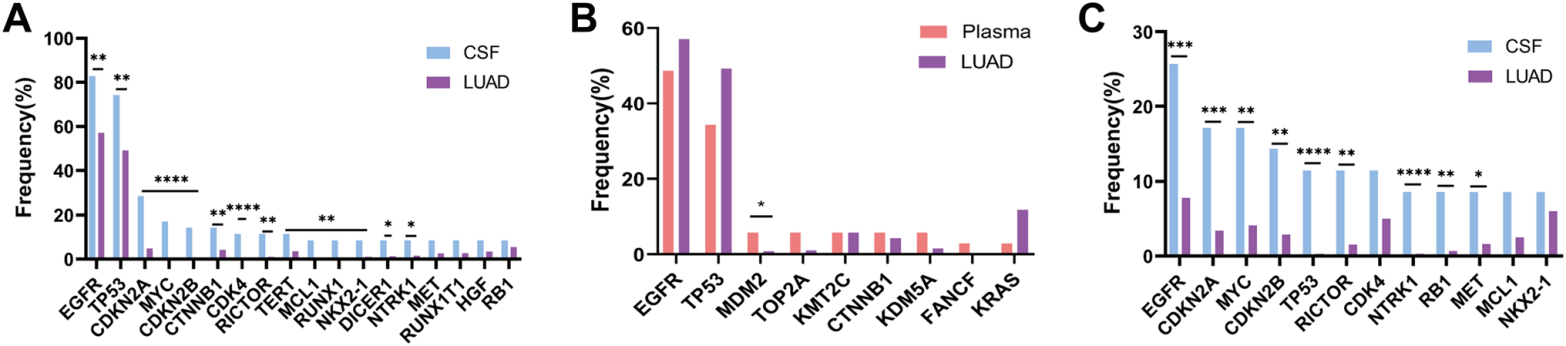
Comparison of mutation frequency of mutated genes between diffrent groups. **A:** Diferences in mutation frequency between CSF and LUAD tissue. **B:** Diferences in mutation frequency between plasma and LUAD tissue. **C:** Diferences in mutation frequency of CNV alterations between CSF and LUAD tissue. CSF: cerebrospinal fluid; LUAD: lung adenocarcinoma; CNV: copy number variation. *P < 0.05, **P < 0.01, ***P < 0.001, and ****P < 0.0001

To assess the potential metastatic mechanism of LM, we compared CSF/plasma samples with LUAD tissue, which was from a China Pancancer study and is a cancer genomic study of a large-scale Asian population [19]. The majority of patients in this study were from China, including 1572 LUAD cases, mostly early cancer specimens (Table S3). There was no significant difference between age and sex compared with our patients. Of note, the mutation frequencies of the *EGFR, TP53, CDKN2A, MYC* and *CDKN2B* genes in CSF were significantly higher than those in LUAD tissue, and these genes were easier to detect in CSF (*P* < 0.05, Fig. 3A). However, the mutation frequencies of *EGFR, TP53 and KRAS* in LUAD tissue were higher than those in plasma, but there were no significant differences. There was a significant increase in *MDM2* mutations in plasma compared with LUAD tissues (*P* < 0.05, Fig. 3B). Subsequent analysis of CNV showed higher frequencies of *EGFR, CDKN2A, MYC, CDKN2B, TP53, RICTOR, NTRK1, RB1* and *MET* in CSF than in LUAD tissue (*P* < 0.05, Fig. 3C). The high occurrence rate of CNV in CSF ctDNA suggested universal genome instability in LM. CSF-specific mutations revealed differences between LM and LUAD tissues. *EGFR, TP53, CDKN2A, MYC* and *CDKN2B* mutations might be potential mechanisms of metastasis.

### Mechanisms of resistance to EGFR-TKIs

We categorized patients with *EGFR* mutations that were detected in CSF, plasma or LUAD tissue into 2 cohorts based on osimertinib administration. In cohort 1 (baseline), a total of 16 patients did not receive osimertinib before the diagnosis of LM with baseline CSF and plasma genotyping. In cohort 2 (resistance), a total of 16 patients progressed after osimertinib and developed LM with CSF and plasma genotyping (Table S4). In cohort 1, icotinib was the most common *EGFR* tyrosine kinase inhibitor (EGFR-TKI). In cohort 2, gefitinib was the most common EGFR-TKI before the application of osimertinib. In cohort 2, 1 patient received osimertinib as first-line therapy, 13 patients as second-line therapy, and 2 patients as third-line treatment. In cohort 1, 87.5% (14/16) of the patients had *EGFR* exon 19 deletion or 21 L858R mutation in CSF, whereas only 50% (8/16) of the patients had these mutations in the matching plasma. In cohort 2, 81.25% (13/16) of the patients had *EGFR* exon 19 deletion or 21 L858R mutation in CSF, whereas only 50% (8/16) of the patients had these mutations in the matching plasma.

The genes varied among the different cohorts **(**Fig. S1**)**. *EGFR* T790M is the most common mechanism of resistance following treatment with 1st- or 2nd-generation EGFR-TKIs. However, in cohort 1 **(**Fig. S1A), an *EGFR*-dependent resistance mechanism of the T790M mutation was found in 2 patients receiving gefitinib or icotinib in plasma but not CSF. *TP53 and CDKN2A* were the most common *EGFR*-independent resistant mutations. In addition, alterations in the *CDK4, CDKN2A, CDKN2B,* and *MYC* gene*s* were identified in CSF. In cohort 2 **(**Fig. S1B), a total of 7 patients (43.75%) had *EGFR* CNV detected in CSF but not plasma after osimertinib administration. Interestingly, alterations of *EGFR* exon 20 T790M, exon 18 G719A and exon 21 insertion were found to occur simultaneously in one patient. However, we found *EGFR*-independent resistance mechanisms of *CDKN2A, CDK4, MET, KRAS,* and *PIK3CA* mutations and *NTRK1* CNV in 5 patients. We did not perform similar histologic and phenotypic transformation assessments on the tissue samples to determine whether there was SCLC transformation due to condition limitations.

### Prognostic factors of LM

**Fig. 4:**
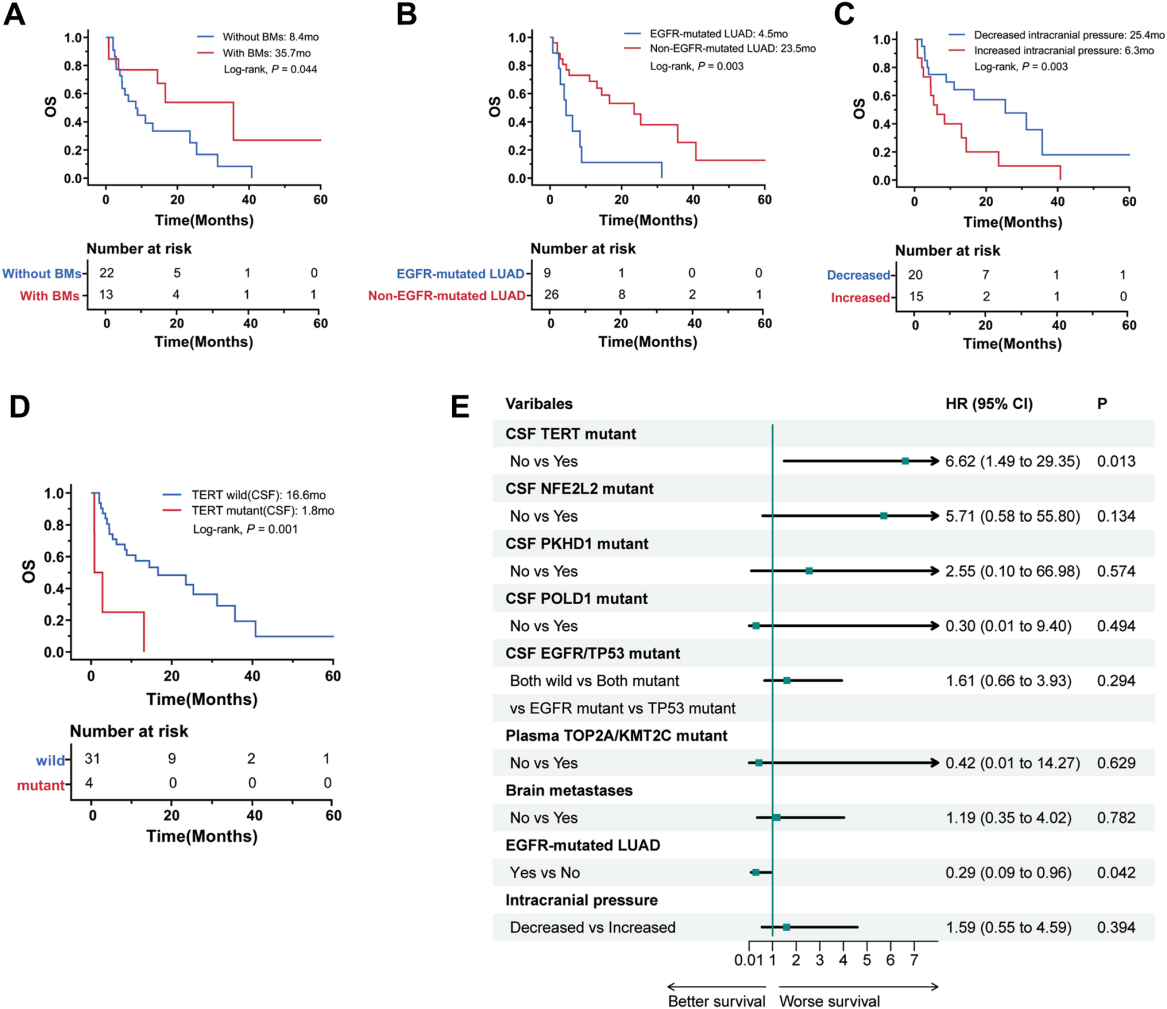
Genetic alterations and clinical features associated with OS. Kaplan‒Meier analysis of OS in patients, **A:** With and without BMs. **B:** EGFR-mutated LUAD and non-EGFR-mutated LUAD. **C:** Decreased intracranial pressure and increased intracranial pressure. **D:** With and without TERT mutation in CSF. **E:** Multivariate Cox proportional hazards regression analysis, EGFR-mutated LUAD and CSF TERT mutant remained independent survival factors. OS: overall survival; EGFR: epidermal growth factor receptor; BMs: brain metastases; CSF: cerebrospinal fluid; TERT: telomerase reverse transcriptase

To further explore the survival prognostic factors of different patients, patient characteristics and various genes were included for interactive survival analysis. The median OS was 14.4 months, and the median time interval between diagnosis of primary tumours and LM was 21.5 months. Patients with BMs, *EGFR*-mutated LUAD and decreased intracranial pressure were associated with longer median OS (BMs, 35.7 months vs. 8.4 months, *P* = 0.044; *EGFR*-mutated LUAD, 23.5 months vs. 4.5 months, *P* = 0.003; decreased intracranial pressure, 25.4 months vs. 6.3 months, *P* = 0.031, Fig. 4A–C). Regardless of whether the enhanced brain MRI was positive, patients with negative CSF cytology still survived (median OS not reached, median follow-up 18.2 months) and survived significantly longer than those with positive CSF cytology (median OS 11.1 months, *P* = 0.035, Fig. S2A). This suggested that floating tumour cells in CSF may lead to a poorer survival prognosis. Based on the genotyping of CSF, the Kaplan‒Meier method revealed that telomerase reverse transcriptase (*TERT*), *NFE2L2, PKHD1* and *POLD1* mutations were associated with shortened median OS (*TERT*, 1.8 months vs. 16.6 months, *P* = 0.001; *NFE2L2*, 2.6 months vs. 14.4 months, *P* = 0.003; *PKHD1*, 4.5 months vs. 16.6 months, *P* = 0.044; *POLD1*, 3.6 months vs. 14.4 months, *P* = 0.032, Fig. 4D, S2B-D).

The co-occurrence of gene mutations may influence prognosis. Therefore, we found that patients with concurrent *EGFR* and *TP53* mutations or concurrent *EGFR* and *TP53* wild-type had a longer median OS than those with *TP53* mutations (*EGFR* and *TP53* mutation vs. *TP53* mutation, 16.6 months vs. 3.6 months, *P* = 0.003; *EGFR* and *TP53* wild-type vs. *TP53* mutation, NA vs. 3.6 months, *P* = 0.049, Fig. S2E). The genetic profile of plasma was also analysed, and *TOP2A* and *KMT2C* mutations indicated lower median OS (*TOP2A*, 3.6 months vs. 14.4 months, *P* = 0.034; *KMT2C*, 3.6 months vs. 14.4 months, *P* = 0.034, Fig. S2G/H). Then, we compared the OS of different *EGFR* mutation states in CSF and plasma, respectively, but no significant difference was found (Fig. S2F/I).

To assess the potential biomedical importance of genetic mutations, we examined their correlations with clinical variables. In further multivariate Cox proportional hazards regression analysis, patients with *EGFR*-mutated LUAD (*P* = 0.042, 95% CI 0.09–0.96 months, Fig. 4E) had a higher median OS, and CSF *TERT* mutation (*P* = 0.013, 95% CI 1.49–29.35 months) indicated a lower median OS. This result suggested that *EGFR-*mutated LUAD and CSF *TERT* mutation remained independent survival factors after adjusting for other patient characteristics.

### CSF ctDNA guided the treatment of LM

Finally, in a case presentation, dynamic changes in CSF ctDNA during the treatment of LM were revealed **(**Fig. S3A, B). In a case presentation, the female patient had a headache for 3 months and underwent a LUAD resection 3 years ago. The patient was diagnosed with LM and BMs because of meningeal enhancement and insular lobe metastatic tumours on brain MRI and malignant cells detected by CSF cytology (CSF1). The patient received gefitinib and antiangiogenic therapy for LM based on *EGFR* p.L858R detected in CSF.

Nine months later, her Karnofsky performance scale (KPS) and Eastern Cooperative Oncology Group performance score (ECOG PS) progressed dramatically, and CSF ctDNA showed a higher frequency of *EGFR* p.L858R. Considering the progression, the targeted therapy was changed to osimertinib 80 mg once a day. Twenty-two months later, she returned with worsening headaches, dizziness, and back pain, which was associated with worse KPS and ECOG PS. CSF cytology revealed a large number of tumour cells (CSF3). The genetic profiles of CSF ctDNA demonstrated *EGFR*-independent resistance mutations **(**Fig. S3B). Unfortunately, the mutation was never detected in plasma. Moreover, a double dose of osimertinib (160 mg) was administered due to changes in the driver genes of CSF ctDNA. It was suggested that CSF ctDNA plays a potential role in the diagnosis and monitoring of LM.

## Discussion

Liquid biopsy is an alternative method to detect mutations in patients if tumour samples are not available. For LM patients, CSF and plasma can be easily obtained. In this research, we characterized the genomic alteration of ctDNA in the CSF and compared it to matched plasma samples. In our study, we revealed that CSF genetic profiles had a unique role in identifying patients with LUAD with LM and that plasma genetic profiles failed to do so. At the time of initial diagnosis of LM, more abundant genotypes were detected in CSF than in plasma in addition to driver mutations. Moreover, we demonstrated that CSF ctDNA has a significantly higher detection rate than plasma. This result is consistent with a previous study that showed that CSF ctDNA exhibits a more comprehensive genetic landscape of CNS metastases than plasma [14, 16, 17, 20]. In previous studies, scholars have compared the detection rate of *EGFR* and found that it is higher in CSF than in plasma. LM is more common in patients with *EGFR* mutations than in those with wild-type *EGFR* [4]. Moreover, in *EGFR*-mutated patients, the detection rate of *EGFR* was 67.6–100% in CSF and 36.4–73.1% in plasma [16]. In our research, *EGFR* was the most frequently mutated gene in CSF, accounting for 82.86%, which was higher than that in matched plasma. This is also consistent with Li’s findings [21], suggesting that patients with *EGFR* mutations may be more susceptible to BMs. In short, the available data revealed heterogeneous genetic profiles between CSF and plasma, with good concordance in driver mutations.

In TRACERx research, subclonal whole genome doubling (WGD) was detected in 29% of lung tumours. These data demonstrate that WGD and copy number heterogeneity were associated with shorter disease-free survival and distant metastases, respectively. WGD and copy number instability are important factors of relapse in NSCLC patients, which guide the evolution of clinical cancer [22]. Numerous CNVs were identified in CSF ctDNA, and they were significantly more than those in plasma in our study. Several studies have indicated that CNVs are enriched in the CSF of CNS metastases [14, 23]. Therefore, we can speculate that CNVs in CSF may cause distant metastasis of tumours and is a major type of mutation that causes LM, exhibiting a difference relative to plasma.

Studies have demonstrated the genetic heterogeneity between the original tumour and the metastatic lesions in the same patient [24]. Genetically distinct subclones of the primary tumour result from somatic evolution of the tumour genome and thus have distinct biologic properties and therapeutic individualization. When tumour cells metastasize, they escape from the primary site, spread and proliferate in secondary locations, and can also evade immune surveillance, eventually forming metastatic lesions. This may lead to the introduction of considerable genomic heterogeneity between the final metastatic cell and the primary cancer [25]. It has been unclear to what extent the genotyping of LMs differs from the genotyping of primary cancers. Previous research demonstrated that overexpression of *MYC, MMP13* or *YAP*, which are enriched for focal amplification in BMs, can each contribute to brain metastasis formation. *TP53, CDKN2A*, and *TERT* are abundant in a variety of metastatic cancers. While *TP53* mutations are strongly associated with genetic instability, *CDK2NA* and *TERT* play a key role in regulating cell proliferation, and both pathways are frequently perturbed in metastatic tumours. In conclusion, these mutated genes may disturb pancancer hallmarks of tumorigenesis, hence improving the aggressiveness of the tumour [26, 27]. To further investigate the importance and evolutionary process of LM genetic alterations, we compared CSF/plasma samples with primary tumour samples. The *EGFR, TP53, MYC, CDKN2A* and *CDKN2B* genes in CSF were significantly higher than those in LUAD tissue. Therefore, genomic characterization of the CSF of patients with LM represents a feasible strategy to find a potential method for the detection of metastasis.

This conclusion was reached in the study of Nanjo et al. that the T790M mutation was less frequent in leptomeningeal than in extracranial specimens by biopsy of patients with lung cancer tissue and leptomeningeal metastases [28]. This study confirmed this idea. After the patients became resistant to 1st- or 2nd-generation EGFR-TKIs, the T790M mutation was identified in plasma but not in CSF, which may be due to the differential expression of the T790M mutation in CNS and extra-CNS lesions.

In the study by Zheng et al., *EGFR* C797S mutation, *MET* dysregulation, and *TP53* plus *RB1* co-occurrence were possible resistance mechanisms of LM in the progression of osimertinib of CSF in NSCLC [29]. Unfortunately, the C797S mutation was not found in our study of cohort 2 because the lack of sufficient samples hindered further discussion. The mechanisms of resistance to osimertinib progression in LM patients may be found in the CSF, such as *EGFR* CNV. Furthermore, another study also revealed that *EGFR* amplification is the resistance mechanism associated with EGFR-TKIs [30]. From the AURA3 trial, *EGFR* mutation was one of the most common acquired resistance mechanisms detected, followed by *MET* amplification [31]. In cohort 2, we found that *EGFR* CNV occurred in 7 patients and *MET* mutations in 3 patients after the diagnosis of LM, which partly accounted for the progressive disease of LM. In our study, we found cell cycle pathway alterations (*CDKN2A, CDKN2B, CDK4,* and *CDK6*) after osimertinib administration. In previous studies, altered cell cycle genes were found to be possibly involved in the mechanism of resistance to osimertinib as the 1st- or 2nd-line therapy [32]. *PIK3CA* amplification or mutations promote tumour infiltration and activate the PI3K/AKT/mTOR pathway, which suggests that PI3K/AKT/mTOR pathway activation might be related to resistance to 3rd-generation EGFR-TKIs [32, 33]. Similar to this report, in our 2 cohorts, the *PIK3CA* mutation was present in the osimertinib-resistant cohort but not in the osimertinib-naive cohort.

Tissue biopsy of the primary tumour to determine the presence of SCLC transformation is also one of the resistance mechanisms to osimertinib [34]. However, the lack of matched primary cancer tissue genetic profiles limits further clarification of the results. The discovery of these important mechanisms of acquired resistance to EGFR-TKIs could facilitate precise treatments for such patients after disease progression.

In our study, a multivariate analysis indicated that the presence of *EGFR*-mutated LUAD was an independent favourable predictor of survival, whereas *TERT* mutation in CSF was an independent predictor of poor survival after excluding other confounding factors. Patients with advanced LUAD who harboured *EGFR* mutations had significantly longer OS than those without *EGFR* mutations after treatment with EGFR-TKIs [35]. Suda et al. reviewed that the better prognosis of patients with *EGFR* mutations may be related to the use of EGFR-TKIs [36]. This has been confirmed by other studies showing that EGFR-TKIs after LM diagnosis were independent favourable predictors of survival [37]. There is no doubt that all 26 patients with *EGFR*-mutated LUAD were treated with EGFR-TKIs before the diagnosis of LM. The *TERT* gene represents a ribonucleoprotease that is essential for the replication of chromosome termini and telomere elongation in eukaryotes. The study suggests that targeting *TERT* promoter (*pTERT*) mutations may serve as a viable approach for cancer therapy [38, 39]. Previous studies have suggested that *TERT* mutations are associated with a poor prognosis in tumours, such as thyroid malignancies, melanoma and gliomas [40–43]. Yang et al. found that *TERT* mutations were detected in 11% of patients with NSCLC, and *TERT* mutation carrier status was an independent risk factor for poor prognosis [44]. Likewise, *TERT* mutations were found in the CSF of 11.43% of patients with LM with LUAD, and these patients had a worse prognosis in our study. This had not been reported to be associated with survival in LM. Thus, we believe that TERT mutation may have clinical value as a potential biomarker for disease monitoring [45, 46].

In the previous literature, *TP53* mutation and *EGFR/TP53* comutation have been considered poor prognostic factors in LUAD patients [47]. In our research, patients with *TP53* mutations in CSF showed shorter OS than those in the other groups (*P* < 0.05). Dual *EGFR/TP53* mutation was associated with inferior OS compared with dual *EGFR/TP53* wild-type, although these results were not statistically significant. In this study, *CDKN2A* was common in CSF samples, accounting for 28.6%, regardless of prognosis. This is consistent with the findings of Yang et al. [48].

In the CSF circulation, disseminated cancer cells can float freely within the CSF or attach to the meninges and can be captured by CSF cytological examination or appear as linear or nodular enhancement on MRI [49]. Remsik et al. found that floating cells were more invasive in vivo than adherent or mixed cells in a mouse model, which was further manifested by the rapid development of neurological symptoms and reduced survival. Remarkably, they found that patients diagnosed with positive CSF cytology only demonstrated substantially diminished survival after LM diagnosis through clinical case collection [50]. Additionally, in the current study, patients with negative CSF cytology still survived and survived significantly longer than those with positive CSF cytology, regardless of whether the enhanced brain MRI was positive.

Finally, we demonstrated with a specific case that dynamic changes in CSF ctDNA at different stages could better predict intracranial tumour responses and track clonal evolution in LM patients.

There are several limitations in our study. First, this was a retrospective study with a small sample size. Second, matched primary lung cancer tissues of the patients were unavailable, and the NGS data were obtained from CSF- or plasma-derived ctDNA without the analysis of matched tumour tissue DNA. Third, there is a lack of observation of CSF tumour markers, and we will continue this study in future observations. However, this is still a rare study that involved exploration of exactly matched CSF and plasma genetic information and analysis of survival prognosis in LUAD patients with LM.

## Conclusions

In conclusion, our findings demonstrate that CSF is a more sensitive and reliable liquid biopsy medium than plasma for LM. CSF ctDNA could provide a more comprehensive genetic landscape of LM, which reveals the potential metastasis-related mechanisms of malignant tumours and the resistance mechanisms to EGFR-TKIs and guides clinical strategies. In addition, *EGFR*-mutated LUAD was associated with better OS, and CSF *TERT* mutation was associated with poorer OS. These indicators may have clinical value as potential novel biomarkers for disease monitoring.

### Supplementary Information

Below is the link to the electronic supplementary material.

Supplementary file1 (TIF 16644 KB)

Supplementary file2 (TIF 21739 KB)

Supplementary file3 (TIF 14230 KB)

Supplementary file4 (DOCX 14 KB)

Supplementary file5 (DOCX 14 KB)

Supplementary file6 (DOCX 14 KB)

Supplementary file7 (DOCX 13 KB)

## Data Availability

The human sequence data from patients are not publicly available due to restrictions on participant privacy. The data that supports the findings of this study are available on reasonable request from the corresponding authors. The China Pan-cancer study (https://www.cbioportal.org/study/summary?id=pan_origimed_2020) was downloaded from cBioPortal.

## Abbreviations

BBB: Blood–brain barrier
BMs: Brain metastases
CNV: Copy number variation
CNS: Central nervous system
CSF: Cerebrospinal fluid
ctDNA: Circulating tumour DNA
EGFR: Epidermal growth factor receptor
EGFR-TKI: EGFR-tyrosine kinase inhibitor
LUAD: Lung adenocarcinoma
LM: Leptomeningeal metastases
MRI: Magnetic resonance imaging
NGS: Next-generation sequencing
NSCLC: Non-small cell lung cancer
OS: Overall survival
