# Supplementary figures and images for: Cerebrospinal fluid circulating tumour DNA genotyping and survival analysis in lung adenocarcinoma with leptomeningeal metastases
Source: J Neurooncol. 2023 Oct 28;165(1):149–60. doi: 10.1007/s11060-023-04471-8 (PMC10638181; doi:10.1007/s11060-023-04471-8)

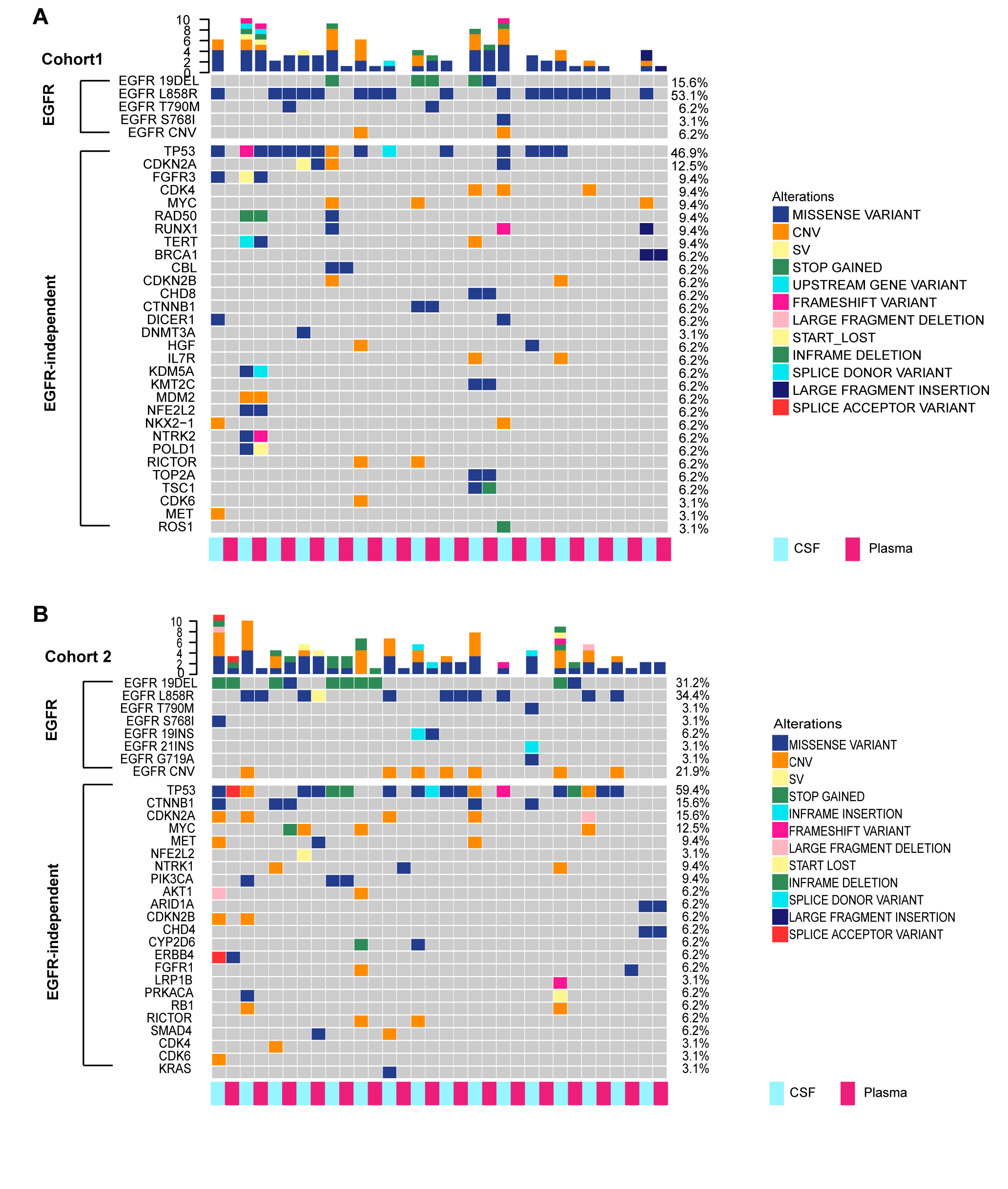

Supplement: Supplementary file 1 — Supplementary file1 (TIF 16644 KB) [file 11060_2023_4471_MOESM1_ESM.tif]

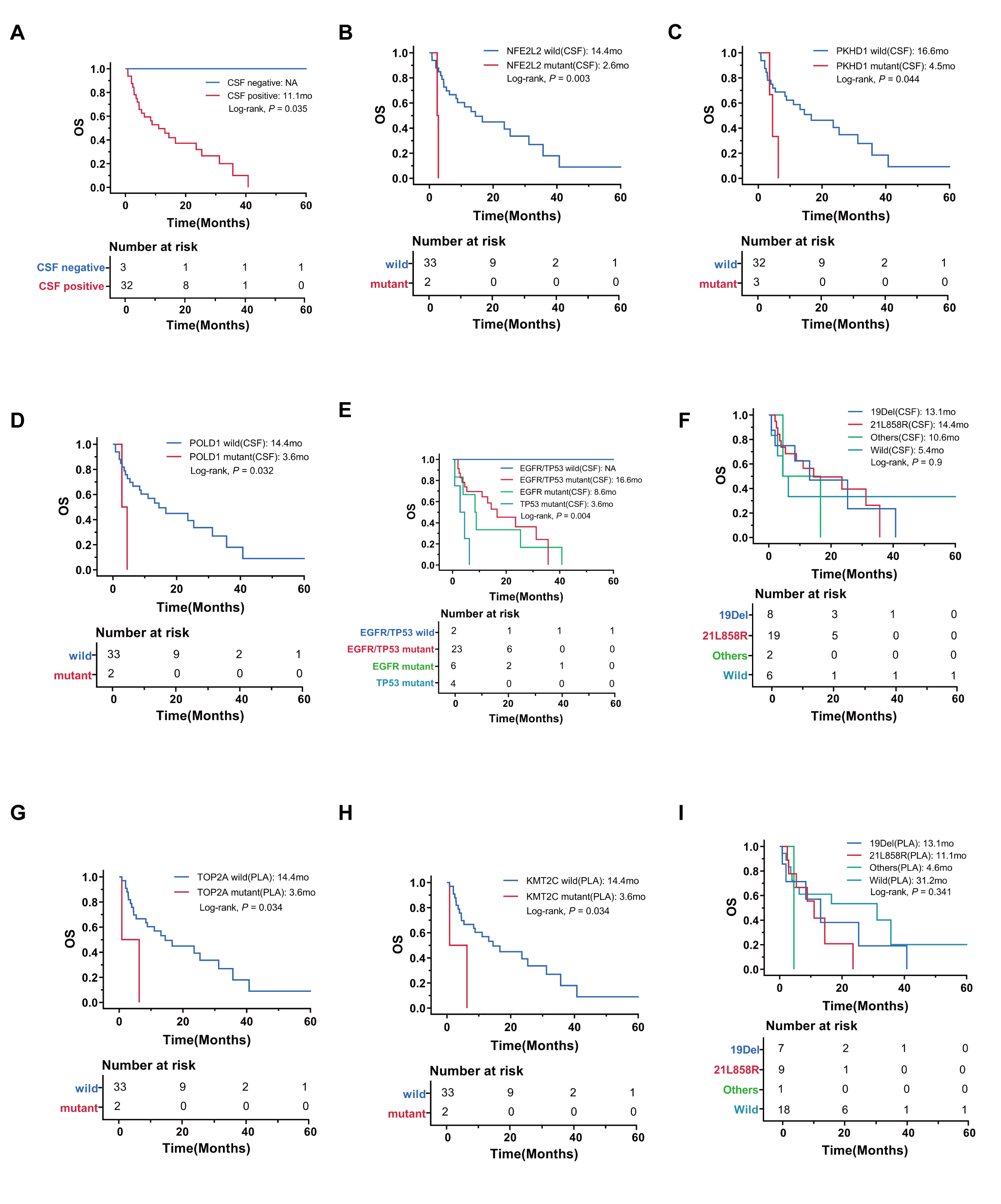

Supplement: Supplementary file 2 — Supplementary file2 (TIF 21739 KB) [file 11060_2023_4471_MOESM2_ESM.tif]

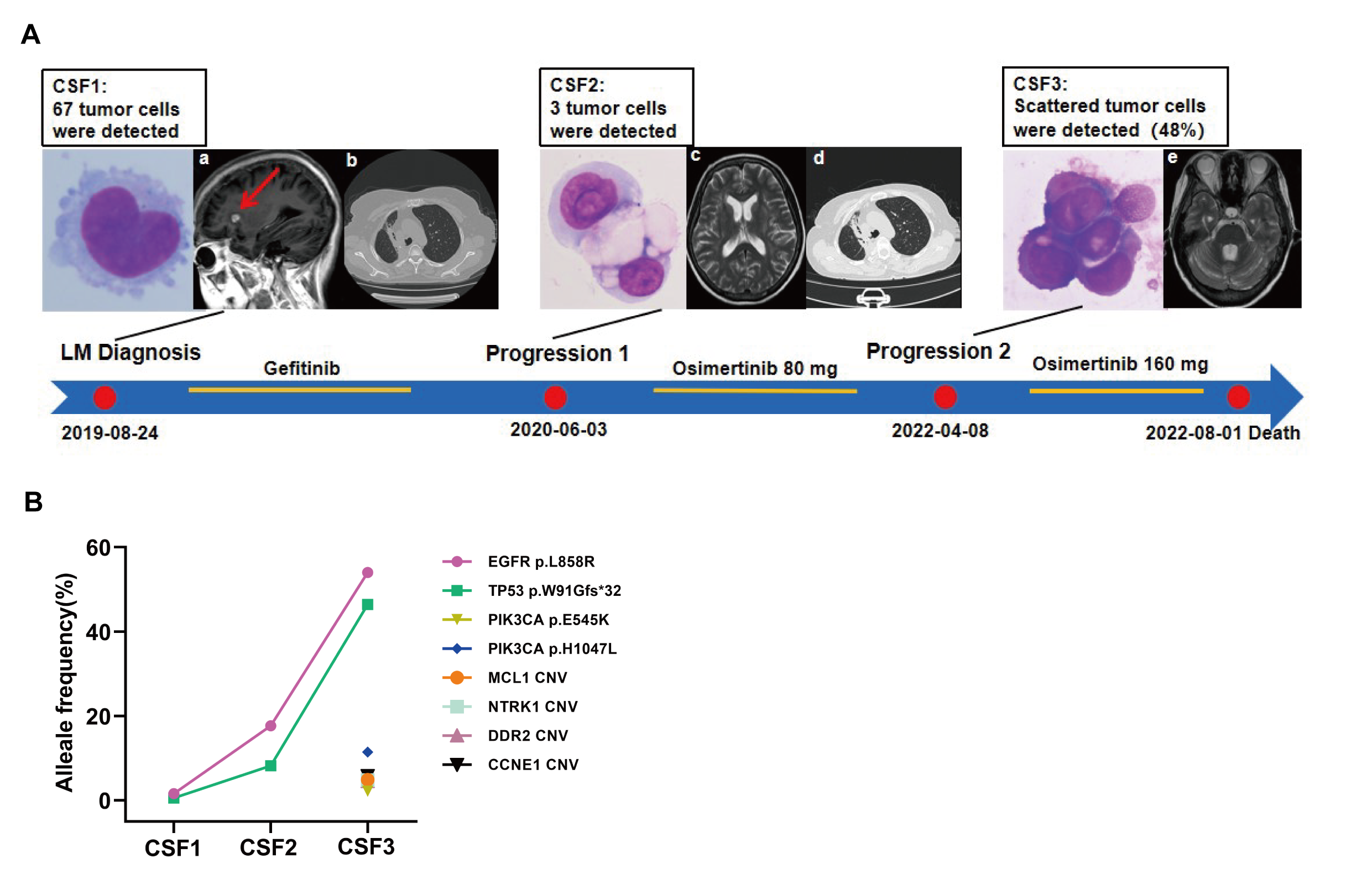

Supplement: Supplementary file 3 — Supplementary file3 (TIF 14230 KB) [file 11060_2023_4471_MOESM3_ESM.tif]
