# Supplementary material for: Cerebrospinal fluid circulating tumour DNA genotyping and survival analysis in lung adenocarcinoma with leptomeningeal metastases
Source: J Neurooncol. 2023 Oct 28;165(1):149–60. doi: 10.1007/s11060-023-04471-8 (PMC10638181; doi:10.1007/s11060-023-04471-8)
Supplement: Supplementary file 4 — Supplementary file4 (DOCX 14 KB) [file 11060_2023_4471_MOESM4_ESM.docx]

**Table S1** 139 lung cancer-associated genes

| AKT1 | AKT2 | AKT3 | ALK | APC | AR |
| --- | --- | --- | --- | --- | --- |
| ARAF | ARID1A | ARID2 | ASXL1 | ATM | ATR |
| ATRX | AXL | BCL2L11(BIM) | BRAF | BRCA1 | BRIP1 |
| BTK | CD274(PD-L1) | CD74 | CDA | CDH1 | CDK4 |
| CDK6 | CDK8 | CDKN1B | CDKN2A | CDKN2B | CHEK2 |
| CREBBP | CTNNB1 | CYLD | CYP2B6 | CYP2C19 | CYP2D6 |
| CYP3A4 | CYP3A5 | DDR2 | DHFR | DNMT3A | DPYD |
| EGFR | ERBB2(HER2) | ERBB3 | ERBB4 | ERCC1 | ERCC2 |
| ERCC4 | FAT1 | FBXW7 | FGFR1 | FGFR3 | FLT4(VEGFR3) |
| FRG1 | GATA4 | GNAS | GRIN2A | GSTM1 | GSTP1 |
| GSTT1 | HDAC9 | HGF | HRAS | IDH1 | IDH2 |
| JAK1 | JAK2 | KDR(VEGF2) | KEAP1 | KIT | KMT2A(MLL) |
| KMT2C | KMT2D(MLL2) | KRAS | LRP1B | LZTR1 | MAP2K1(MEK1) |
| MAP2K2(MEK2) | MED12 | MET | MLH1 | MTHFR | MTOR |
| MYC | NBN | NF1 | NF2 | NFE2L2 | NOTCH1 |
| NQO1 | NRAS | NTRK1 | NTRK3 | PBRM1 | PDCD1(PD1) |
| PDCD1LG2(PD-L2) | PDGFRA | PDGFRB | PIK3CA | PIK3CD | PIK3R1 |
| PTEN | PTPN11 | QKI | RAF1 | RB1 | RECQL4 |
| RELN | RET | RHOA | RICTOR | ROS1 | SBDS |
| SDC4 | SETD2 | SF3B1 | SLC34A2 | SMAD2 | SMAD3 |
| SMAD4 | SMARCA4 | SMARCB1 | SOX2 | STAG2 | STAT3 |
| STK11 | TET2 | TGFBR2 | TP53 | TPMT | TSC1 |
| TSC2 | TYMS | U2AF1 | UGT1A1 | VEGFA | WRN |
| XRCC1 |  |  |  |  |  |
