# Supplementary material for: Cerebrospinal fluid circulating tumour DNA genotyping and survival analysis in lung adenocarcinoma with leptomeningeal metastases
Source: J Neurooncol. 2023 Oct 28;165(1):149–60. doi: 10.1007/s11060-023-04471-8 (PMC10638181; doi:10.1007/s11060-023-04471-8)
Supplement: Supplementary file 5 — Supplementary file5 (DOCX 14 KB) [file 11060_2023_4471_MOESM5_ESM.docx]

**Table S2**  Patient demographic and clinical characteristics

| Characteristic | N (%) |
| --- | --- |
| CSF cytologic findings |  |
| Positive | 32 (91.43) |
| Negative | 3 (8.57) |
| Enhancement on Brain MRI |  |
| Positive | 24 (68.57) |
| Negative | 10 (28.57) |
| NA | 1 (2.57) |
| Diagnosis of LM |  |
| CSF cytology Brain MRI |  |
| (+) (+) | 21 (60) |
| (+) (-) | 10 (28.57) |
| (-) (+) | 3 (8.57) |
| (+) NA | 1 (2.86) |
| Presentation of LM |  |
| [Simultaneity](javascript:;) | 2 (5.71) |
| During treatment | 33 (94.29) |
| CSF findings at LM diagnosis |  |
| Intracranial pressure |  |
| ≤200 mm H_2_0 | 20 (57.14) |
| >200 mm H_2_0 | 15 (42.86) |
| CSF WBC level |  |
| ≤5 per mm^3^ | 9 (25.71) |
| >5 per mm^3^ | 26 (74.29) |
| CSF protein level |  |
| ≤50mg/dl | 19 (54.29) |
| >50mg/dl | 16 (45.71) |
| CSF glucose level |  |
| ≤40mg/dl | 12 (34.29) |
| >40mg/dl | 23 (65.71) |
| CSF cl level |  |
| Abnormal (≤120mmol/L) | 15 (42.86) |
| Normal(>120mmol/L or ≤132mmol/L) | 20 (57.14) |

LM: leptomeningeal metastasis; MRI: magnetic resonance imaging; CSF: cerebrospinal fluid.
