# Supplementary material for: Cerebrospinal fluid circulating tumour DNA genotyping and survival analysis in lung adenocarcinoma with leptomeningeal metastases
Source: J Neurooncol. 2023 Oct 28;165(1):149–60. doi: 10.1007/s11060-023-04471-8 (PMC10638181; doi:10.1007/s11060-023-04471-8)
Supplement: Supplementary file 6 — Supplementary file6 (DOCX 14 KB) [file 11060_2023_4471_MOESM6_ESM.docx]

**Table S3** The clinical characteristics of the 1572 LUAD cases from a large-scale Asian population

| Characteristic | N (%) |
| --- | --- |
| Sex |  |
| Male | 782 (49.75) |
| Female | 790 (50.25) |
| Age at diagnosis |  |
| Median (range) | 59 (27-92) |
| <60 | 805 (51.21) |
| ≥60 | 767 (48.79) |
| Smoking status |  |
| Smoker | 408 (25.95) |
| Nonsmoker | 962 (61.20) |
| Unknwon | 202 (12.85) |
| Stage at diagnosis |  |
| 0 | 2 (0.13) |
| Ⅰ | 612 (38.93) |
| Ⅱ | 152 (9.67) |
| Ⅲ | 272 (17.30) |
| Ⅲ-Ⅳ | 1 (0.06) |
| Ⅳ | 532 (33.84) |
| Unknown | 1 (0.06) |
