# Supplementary material for: Cerebrospinal fluid circulating tumour DNA genotyping and survival analysis in lung adenocarcinoma with leptomeningeal metastases
Source: J Neurooncol. 2023 Oct 28;165(1):149–60. doi: 10.1007/s11060-023-04471-8 (PMC10638181; doi:10.1007/s11060-023-04471-8)
Supplement: Supplementary file 7 — Supplementary file7 (DOCX 13 KB) [file 11060_2023_4471_MOESM7_ESM.docx]

**Table S4** The clinical characteristics of 32 patients with EGFR mutation

| Characteristic | Cohort 1^a^, Baseline  N (%) | Cohort 2^b^, Resistance  N(%) |
| --- | --- | --- |
| No. of patients | 16 (50) | 16 (50) |
| EGFR mutation status in CSF |  |  |
| 19DEL | 3 (18.75) | 5 (31.25) |
| 21L858R | 11 (68.75) | 8 (50) |
| EGFR CNV | 2 (12.5) | 7 (43.75) |
| Others^c^ | 1 (6.25) | 3 (18.75) |
| EGFR mutation status in plasma |  |  |
| 19DEL | 2 (12.5) | 5 (31.25) |
| 21L858R | 6 (37.5) | 3 (18.75) |
| EGFR CNV | 0 (0) | 0 (0) |
| Others^d^ | 2 (12.5) | 1 (6.25) |
| EGFR-TKIs before LM^e^ |  |  |
| Gefitinib | 6 (37.5) | 9 (56.25) |
| Icotinib | 8 (50) | 0 (0) |
| Erlotinib | 1 (6.25) | 4 (25) |
| Dacomitinib | 0 (0) | 1 (6.25) |
| Afatinib | 0 (0) | 1 (6.25) |
| Almonertinib | 0 (0) | 1 (6.25) |
| Osimertinib | 0 (0) | 16 (100) |
| Line of osimertinib before LM |  |  |
| First line | - | 1 (6.25) |
| Second line | - | 13 (81.25) |
| Over second line | - | 2 (12.5) |

a: Patients who didn't receive osimertinib before the diagnosis of LM.

b: Patients who progressed after osimertinib and developed LM.

c: Cohort 1: EGFR exon 20 S768I mutation; Cohort 2: EGFR exon 20 S768I mutation, EGFR 19 insertion, EGFR exon 18 G719A/ exon 20 T790M/ exon 21 insertion.

d:Cohort 1: exon 20 T790M; Cohort 2: EGFR 19 insertion.

e: In cohort 1, three patients received at least one EGFR-TKIs, and in cohort 2, fifteen patients received at least one EGFR-TKIs.
